# Functional diversity drives ecosystem multifunctionality in a *Pinus yunnanensis* natural secondary forest

**DOI:** 10.1038/s41598-019-43475-1

**Published:** 2019-05-06

**Authors:** Xiaobo Huang, Jianrong Su, Shuaifeng Li, Wande Liu, Xuedong Lang

**Affiliations:** 1 Research Institute of Resources Insects, Chinese Academy of Forestry, Kunming, 650224 China; 2 Pu’er Forest Ecosystem Research Station, National Forestry and Grassland Administration, Kunming, 650224 China

**Keywords:** Forest ecology, Forest ecology

## Abstract

It is essential to understand how the loss of biodiversity impacts both ecosystem function (EF) and multifunctionality (EMF). Previous studies have mostly focused on predicting how species richness (SR) impacts EMF, while the effect of functional diversity (FD) on EMF remains unclear. Specifically, we know little about the primary functional drivers impacting EMF compared with SR. Therefore, we analysed 8 ecosystem functions within 58 natural secondary forest plots to investigate the effect of FD on both individual EF and EMF. Our results suggest that SR and FD had very significant positive effects on plant phosphorus, soil available phosphorus, and soil total nitrogen. FD explained significantly more variations in these functional responses than SR for individual ecosystem functioning. We also used a multiple threshold approach to test the effect of SR and FD on EMF. We found that FD and SR were positively related to EMF regardless of whether low-level function or high-level function was desired, but FD had a larger effect than SR. Based on the averaging approach, OLS regression, multivariate linear regression model and random forest analysis, we found that SR and FD were both drivers of EMF but that FD had a stronger effect and could explain more variation. As such, we conclude that FD drives ecosystem multifunctionality more than SR.

## Introduction

Biodiversity is crucial for sustaining ecosystem processes and functioning^1^. Biodiversity is declining at an unprecedented rate and will continue to decline over the 21^st^ century^2^. As such, understanding how the loss of biodiversity impacts ecosystem functioning has become an important goal in biology and conservation^3^. The study of the relationship between biodiversity and ecosystem functioning (hereafter, BEF) has been ongoing for over twenty years^4,5^. Many empirical studies have demonstrated that a loss of biodiversity can lead to reductions in ecosystem functioning^6^ and the ecosystem services that provide a multitude of benefits to humans^7,8^. Progress in studies focused on BEF has been spurred with the deepening of understanding, and scientists found that ecosystems can perform multiple functions or services simultaneously^1,9^. Thus, multifunctionality (hereafter, EMF) has been generally accepted in recent years and is defined as “the simultaneous provision of multiple functions”^10^. Because different species have different functional traits, they could contribute different ecosystem functions. Thus, biodiversity might have an important effect on the multifunctionality^11^.

Most experiments conducted on the relationship between biodiversity and ecosystem multifunctionality (hereafter, BEMF) have focused on species richness (hereafter, SR) as the primary metric of biodiversity^1,9–14^ because SR is defined as the number of species and is easily measured. However, some studies suggest that functional diversity (hereafter, FD) is a stronger predictor of ecosystem functioning^15–17^. The effect is most immediate mainly because the effect of FD is the result of interactions between species and their environment relative to the species traits^15^. The reason for FD as a stronger predictor may be due to high FD causing an increase in the utilization efficiency of environmental resources, which can subsequently promote ecosystem productivity and strengthen defences against diseases, insect pests, and disturbances^18,19^.

Developing a better understanding of the links between diversity and ecosystem functioning is especially important in forest ecosystems. Forests provide innumerable benefits for humans, including timber, nutrient and water cycling, and recreational opportunities, among many others^8,13^. Despite this, previous studies on BEMF have mostly focused on experimental grassland ecosystems^1,20–23^. Regarding forests, more attention has been paid to temperate and boreal forests. Most of studies proved that higher biodiversity was necessary to maintain multiple ecosystem functions among different ecosystems, but little attention has been paid to subtropical forests^8,24–26^. We noticed that some scientists, such as Mouillot *et al*.^27^, Valencia-Gómez *et al*.^28^ and Finney & Kaye^29^, conducted many studies on the influence of FD on EMF. These studies found that FD could increase multifunctionality. However, this result was found in grasslands, dry lands or agricultural systems, and we did not identify related studies on forests to date.

**Figure 1:**
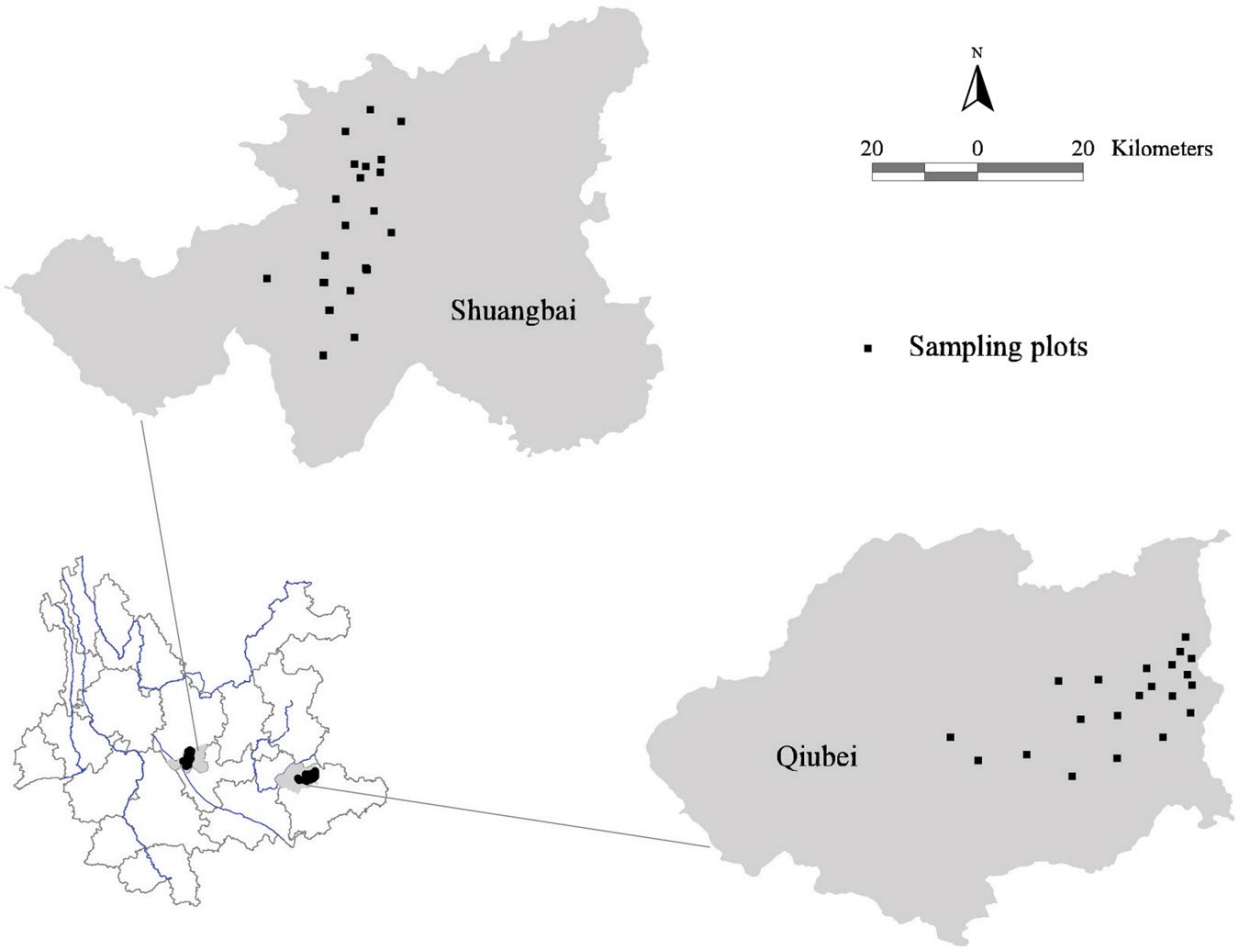
Map showing the location of our study (black circles, *n* = 58).

Here, we seek to help fill this gap using data collected from a *Pinus yunnanensis* forest in southwest China^30^. We used data collected from 58 natural secondary forest plots located in Qiubei and Shuangbai County in Yunnan Province, China (Fig. 1) and examined eight individual ecosystem functions (plant nitrogen, plant phosphorus, soil hydrolysable nitrogen, soil available phosphorus, soil total nitrogen, soil total phosphorus, soil total carbon, and woody plant biomass). Aboveground biomass was the primary key function in the BEMF study^8,31^. In the soil samples, soil total carbon, soil total nitrogen, soil hydrolysable nitrogen, soil total phosphorus and soil available phosphorus represent good substitutions related to carbon (C), nitrogen (N) and phosphorus (P) cycling^9,21^. Nitrogen and phosphorus are two important nutrient elements for plants. Nitrogen mainly determined photosynthetic C fixation and plant productivity^32^. Phosphorus is considered necessary for the storage and reproduction of genetic information and is mainly involved in energy-related processes of living organisms^33^. Regardless of whether nitrogen or phosphorus was limited, plant growth was affected. Plant nitrogen and plant phosphorus are considered nutrient pools in aboveground biomass^21^. We used this unique dataset to address two primary questions: (1) How do SR and FD influence individual ecosystem functions?; (2) Does FD predict EMF better than SR? We expected to identify the relative importance of biotic and abiotic variables on EMF in a *Pinus yunnanensis* natural secondary forest.

## Results

### Effect of biodiversity on individual ecosystem functions

Regarding individual functions, the relationships between SR and plant phosphorus (PP) (*r* = 0.442, *p* < 0.01), soil available phosphorus (SAP) (*r* = 0.331, *p* < 0.05) and soil total nitrogen (STN) (*r* = 0.261, *p* < 0.05) were all significantly positive. SR accounted for 19.7%, 12.3% and 8% of the variation in these variables, respectively (Table 1). The results were similar when analysing FRic. FRic had a significantly positive correlation with PP (*r* = 0.500, *p* < 0.01), SAP (*r* = 0.451, *p* < 0.01) and STN (*r* = 0.310, *p* < 0.05), although the total amount of explained variation was higher than SR in all cases (Table 1). For plant nitrogen (PN), soil hydrolysable nitrogen (SHN), soil total phosphorus (STP), soil total carbon (STC) and woody plant biomass (WPB), there were no significant correlations with SR and FRic (Table 1).

**Table 1:** Relationships among SR, FRic and individual functions in the *Pinus yunnanensis* natural secondary forest based on OLS regression analysis.

Function parameters	SR		FRic	
	*R* ^2^	*P*	*R* ^2^	*P*
PN	0.007	0.524	0.000	0.943
PP	0.197	<0.001	0.244	<0.001
SHN	0.055	0.076	0.064	0.056
SAP	0.123	<0.01	0.213	<0.001
STN	0.080	<0.05	0.107	<0.05
STP	0.023	0.254	0.063	0.058
STC	0.057	0.070	0.057	0.070
WPB	0.000	0.973	0.000	0.900

PN: Plant nitrogen; PP: Plant phosphorus; SHN: Soil hydrolysable nitrogen; SAP: Soil available phosphorus; STN: Soil total nitrogen; STP: Soil total phosphorus; STC: Soil total carbon; WPB: Woody plant biomass.

### BEMF relationships based on the averaging approach

**Figure 2:**
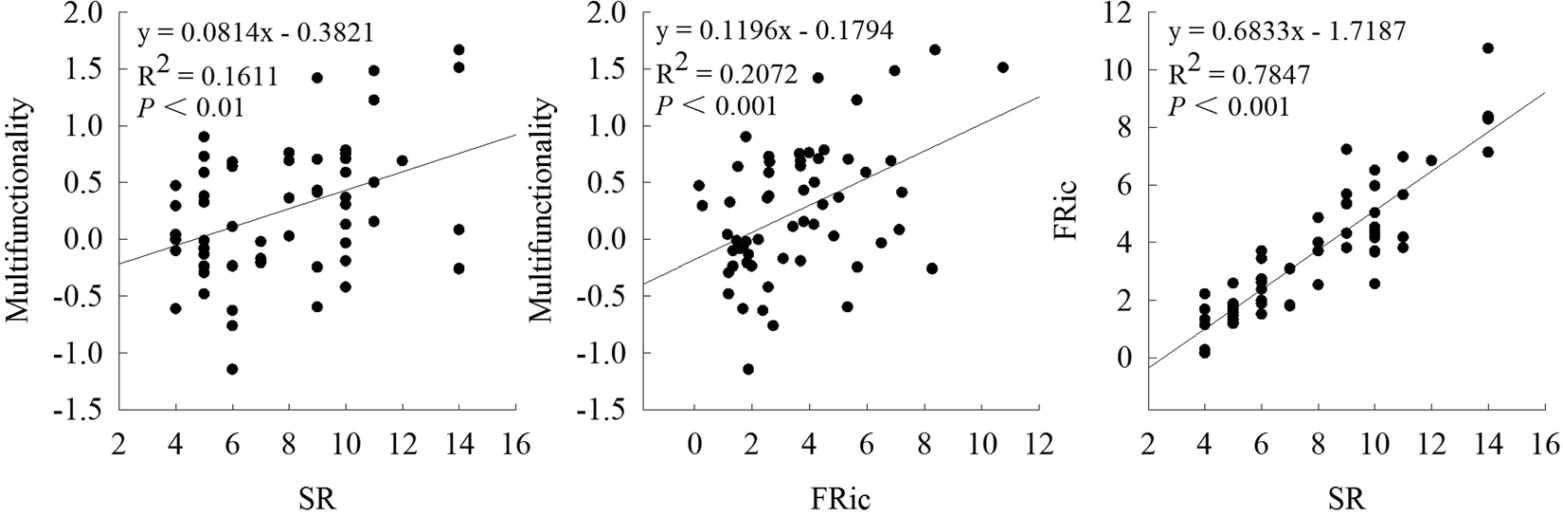
The effects of SR and FRic on EMF (calculated by averaging approach) and the relationship between SR and FRic in the *Pinus yunnanensis* natural secondary forest based on OLS regression analysis. The solid line represents the fitted OLS regression.

Both SR and FRic were related to EMF. SR explained 16.11% of the variation in EMF, while FRic explained 20.72% of the variation. SR was significantly positively correlated with FRic (*R*^2^ = 0.7847, *P* < 0.001) (Fig. 2).

### Effect of SR and FRic on EMF

**Figure 3:**
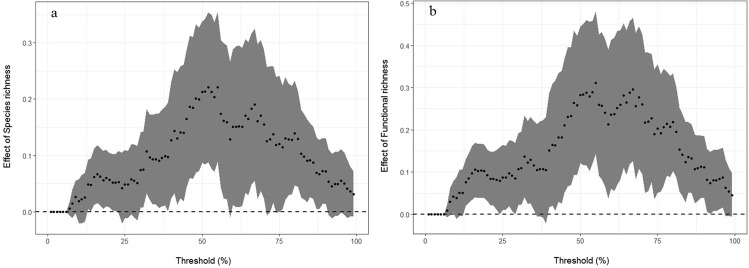
The effect of SR and FRic on EMF. Based on the multiple threshold approach. (**a**) The effect of SR (each increase in SR was associated with the number of functions) for a range of multifunctionality thresholds. (**b**) The effect of FRic (each increase in FRic was associated with the number of functions) for a range of multifunctionality thresholds. The dotted, horizontal line indicates an SR or FRic effect of zero. The points are fitted values, and shading indicates ±1 confidence intervals.

SR had a positive effect on EMF at thresholds between 12% (minimum threshold, *T*_min_) and 91% (maximum threshold, *T*_max_) (Fig. 3a), and FRic had a positive effect on EMF at thresholds between 8% (minimum threshold, *T*_min_) and 97% (Fig. 3b). The relationship between SR and EMF peaked at a threshold of 55% (threshold of maximum diversity effect, *T*_mde_), where each additional species was associated with 0.22 functions provided at levels above the threshold (realized maximum effect of diversity, *R*_mde_) (Fig. 3a). The relationship between FRic and EMF also peaked at a threshold of 55%, but each increase in FRic was associated with 0.31 functions (Fig. 3b). The maximum possible effect of SR (percentage of maximum possible diversity effect, *P*_mde_) for our experiment was 36.75%, and the *P*_mde_ of FRic was 41.85% (Fig. 3).

### Estimation of the relative contribution of the biotic and abiotic factors to EMF

The results of the multivariate linear regression model suggested FRic of the biotic factor had a significant positive effect on EMF. The absolute value of the standardized regression coefficients of FRic (0.6535) was the biggest among these variables, indicating that FRic had the strongest impact on EMF compared with other factors. SR of the biotic factor and the abiotic factors, including soil pH, MAP and MAT, had no significant effect on EMF (Table 2).

**Table 2:** Summary of the results obtained from the multivariate linear regression model showing the integrative effects of biotic factors (SR, FRic) and abiotic factors (soil pH, MAP and MAT) on EMF in the *Pinus yunnanensis* natural secondary forest.

Factors	Standardized regression coefficients	Partial regression coefficients	Std. Error	t value	*P*
FRic	0.6535	0.384	0.068	2.507	0.015*
SR	−0.2738	−0.161	0.054	−1.116	0.269
MAT	−0.0005	−0.0003	3.940	−0.004	0.997
MAP	−0.0886	−0.052	2.735	−0.475	0.637
Soil pH	−0.0286	−0.017	2.868	−0.167	0.868

MAP: Mean Annual Precipitation; MAT: Mean Annual Temperature.

*P < 0.05.

**Figure 4:**
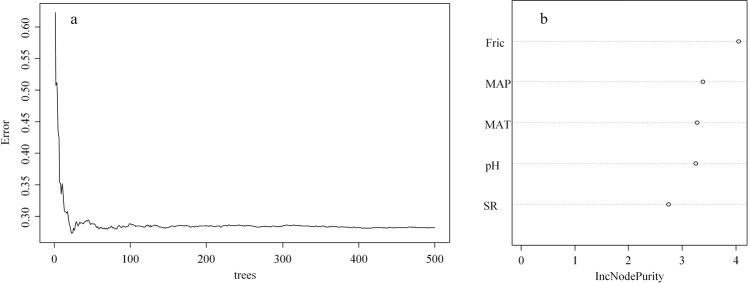
Number of trees (forest) in training stage to meet the minimum error (**a**) and the variable importance obtained by random forest analysis (**b**).

According to random forest analysis, we found that FRic had the highest importance for EMF in all variables. In contrast, SR had the lowest importance among all selected variables. For the abiotic factors, the rank of importance was MAP, MAT, and soil pH (Fig. 4).

### The standardized effect size (SES) value of FRic and eight individual EFs

The above results demonstrated that FRic was the main driver for EMF. For individual EFs, the SES values of FRic and PN, PP, SHN, SAP, STN, STP, and SOC were positive. The order of SES value was PP (3.8224) > SAP (3.4224) > STN (2.3323) > SHN (1.9707) > STC (1.8746) > STP (1.7986) > PN (0.0997). The SES value of FRic and WPB was minimal and negative; the value was −0.0846 (Table 3).

**Table 3:** The standardized effect size (SES) values with 95% confidence intervals of three traits (SLA, specific leaf area; WD, wood density; LA, leaf area), FRic and eight individual ecosystem functions (PN, PP, SHN, SAP, STN, STP, SOC, WPB), and EMF.

Type	Standardized effect size (SES)								
	PN	PP	SHN	SAP	STN	STP	STC	WPB	EMF
SLA	1.9436	3.2469	−0.3391	1.9056	−0.3847	1.3776	−1.0604	−2.1806	1.1563
WD	−1.8480	−2.5130	−0.5980	−1.6881	−0.3373	−3.3879	0.9779	1.3962	−1.8514
LA	2.2799	0.2153	0.0535	0.0234	−0.3679	0.5849	−0.4515	−2.0414	0.1249
FRic	0.0997	3.8224	1.9707	3.4224	2.3323	1.7986	1.8746	−0.0846	3.4408

## Discussion

The primary goal of this work was to determine the relationship between biodiversity and multiple measures of ecosystem function. We found that both SR and FRic were positively correlated with three of the eight potential components of ecosystem functioning: plant phosphorous, soil available phosphorus and soil total nitrogen. Furthermore, these effects were observed when combining different measures and calculating ecosystem multifunctionality. Importantly, in all cases, we found that FRic was a better predictor than SR.

Functional diversity is thought to be declining worldwide^34^. The number of studies about the relationships between functional diversity and ecosystem functioning is increasing. However, the role of functional diversity in EMF has not been fully explored. This notion is particularly true for forest communities, which have been less well studied within the context of BEMF. In this study, we found that several individual ecosystem functions, such as plant phosphorus, soil available phosphorus and soil total nitrogen, were sensitive to changes in SR and FRic. For the two metrics of biodiversity, SR and FRic, the relationship between them and the individual ecosystem functions was relatively consistent. However, FRic could explain more variations in these three ecosystem functions than SR. These results were consistent with most previous studies^17,35,36^. It is worth mentioning that SR and FRic had no effect on woody plant biomass in this study, and the result was not consistent with previous conclusions of BEF studies^37^. This difference could be due to the structure of the *Pinus yunnanensis* natural secondary forest. In this forest type, *Pinus yunnanensis* held an absolutely dominant position relative to other species^30^. *Pinus yunnanensis* contributed 91.73% of the biomass in our study, so increasing or decreasing one species might not have a significant effect on the biomass. When considering multiple ecosystem functions simultaneously, biodiversity likely has a stronger effect^22^. In our study, this conclusion was confirmed regardless of whether SR or FRic served as the metric of biodiversity.

Our results also indicate no strong trade-offs between different functions in the *Pinus yunnanensis* natural secondary forest (Table S2). Normally, trade-offs between different functions are commonplace within local communities^1,20,38^. However, at larger spatial scales, heterogeneity in a community composition causes different parts of the landscape to provide different ecosystem functions under the precondition that different species provide different ecosystem functions^1,13,20^. In theory, this implies that all ecosystem functions could be maximized simultaneously if information on their causal relationships is known. These results were consistent with the results of van der Plas *et al*.^26^, who found that trade-offs were weak among different functions at larger scales.

We used the multiple threshold approach to evaluate whether and when SR or FRic were important drivers of EMF. We obtained a complete picture of how SR or FRic drove EMF by combining some metrics; FRic had an obvious positive effect on EMF at thresholds between 8% (minimum threshold) and 97% (maximum threshold). This range was wider than that for SR. Byrnes *et al*.^10^ indicated that if the *T*_min_ was low, the *T*_max_ was high, and both the *T*_mde_ and the *R*_mde_ remained high, diversity would have a strong effect on EMF. Compared with that observed for SR, these parameters all had the strongest effect at a moderate threshold (*T*_mde_ = 55%). However, *R*_mde_ and *P*_mde_ values of FRic were greater than those of SR; therefore, our results at least showed that the effect of FRic on EMF was greater than that of SR.

In the multivariate linear regression model and random forest analysis, we all found that FRic was the main driver in BEMF. Our result is consistent with previous studies^27–29^. Compared with taxonomic diversity for grassland, dry land, agricultural or subtropical forest ecosystems, functional diversity is the strongest predictor of EMF and has a positive effect. For EFs, in our study, functional diversity has a positive effect with the expectation of WPB (Table 3) because functional diversity is based on functional traits. Functional traits are the key mechanism by which single species and groups of species affect ecosystem functions^39^.

It should be noted that there is an inevitable connection between SR and FRic^40^. In this study, we observed a strong correlation between these variables (*P* < 0.001, *R*^2^ = 0.7847). Because FRic is influenced by SR, it is somewhat difficult to tease apart the fraction of variation that is uniquely explained by functional diversity^41^. This problem could potentially be resolved using experimental studies in combination with variance partitioning analyses.

## Conclusions

We found that 3 of 8 ecosystem functions, PP, SAP and STN, were strongly correlated with SR and FRic. However, FRic could explain more variations in these three ecosystem functions. Using a multiple threshold approach, we proved that the effect of FRic on EMF was greater than the effect of SR. In addition, we found no strong trade-offs among ecosystem functions in the *Pinus yunnanensis* natural secondary forest. Thus, all ecosystem functions may be maximized simultaneously. Moreover, our findings provide strong evidence that FRic drives EMF more than SR. For ecosystem managers, our findings are useful. Functional diversity was based on multiple functional traits. Thus, to increase EMF, the managers could start with species assemblages and chose species with functional traits as different as possible. To conserve biodiversity, we reinforce the view that if conserving or promoting biodiversity is the target in an ecosystem, ensuring a moderate level of EMF is at least a precondition^25^.

## Materials and Methods

### Plot selection and sampling

Field surveys were conducted in April 2015. In total, 58 forest plots measuring 20 × 20 m were established under the most representative vegetation over an extensive area (approximately 9, 000 km^2^). Across all plots, the mean annual temperature ranged from 15.7 to 18.2 °C, the mean annual precipitation ranged from 954 to 1202 mm, and the altitude ranged from 1065 to 2125 m. For our study, the number of plots was sufficient to statistically detect the relationship between biodiversity and ecosystem functioning^42^. We selected plots with a gradient of plant species to detect the relationship between biodiversity and ecosystem functions and tried to minimize other confounding factors, for example, selected the forest of the same age, undisturbed soil and plant community structure. Within the plots, we collected information on species identity, height, DBH, and spatial location for all individuals >1 cm DBH. For each forest plot, we obtained the MAT and MAP data from ClimateAP (University of British Columbia, Vancouver, British Columbia, Canada) developed by Wang *et al*.^43^.

For all species, we also collected 50–100 sun-exposed mature leaves from the middle or upper part of the trees from five to ten individuals. All samples were carefully placed into paper bags and marked. We then measured leaf morphological characteristics using a laser area metre (LI-COR 3100C Area Meter, LI-COR, USA). The leaf area of *Pinus yunnanensis* was estimated as a cylinder, so we measured the diameter (*d*) and the length (*L*) by a Vernier calliper (precision: 0.02 mm). The leaf area of *Pinus yunnanensis* was calculated with the following formula: LA = 2π$\sqrt{3}$/9·*dL*^44^.

All leaf samples were dried for 72 h at 60 °C, and the dry weight for each leaf was measured immediately with an electronic balance (precision: 0.0001 g). Afterwards, all samples were ground to a fine powder using a ball mill (NM200, Retsch, Haan, Germany) to measure plant N and P content. We also obtained 5–10 branches (1 cm ≤ DBH ≤ 2 cm) from some shrub species (DBH ≤ 5 cm). We used the water replacement method to measure branch volume after removing the peel. Then, all branches were dried for 72 h at 103 °C and subsequently weighed. The density of the branches was calculated as the ratio of the dry weight to the volume. For trees and shrubs >5 cm DBH, we sampled the wooden core by the growth cone. The volume was measured using the diameter and the length of the wooden core, and the wood density was calculated as the ratio of the dry weight to the volume of the wooden core. All functional traits were measured following standardized protocols^45^. For each plot, we collected 5 soil samples (0–20 cm) and mixed them evenly. The soil samples were sieved by a 2-mm mesh and air-dried for one month prior to physiochemical analyses.

### Defining biodiversity

We used species richness (SR) and functional richness (FRic) as our measures of biodiversity. To correspond to species richness, FRic was used to measure functional diversity. We chose three commonly used plant functional traits to calculate functional diversity, including leaf area (LA, mm^2^), specific leaf area (SLA = leaf area/dry weight, mm^2^·mg^−1^), and wood density (WD = wood dry weight/wood volume, g·cm^−3^). These traits were expected to be related to the acquisition and utilization of resources in plants, including trees^46^.

### Measurement of individual ecosystem functions

We measured eight ecosystem properties or characteristics (used as proxies of functions) linked to total nutrient pools, nutrient cycling, and biological productivity. These ecosystem functions were as follows: plant nitrogen, plant phosphorus, soil hydrolysable nitrogen, soil available phosphorus, soil total nitrogen, soil total phosphorus, soil total carbon, and woody plant biomass. These ecosystem functions were consistent with previous studies^21,47^. Soil total carbon, plant nitrogen and soil total nitrogen were measured by combustion in a CHN elemental analyser (2400II CHN elemental analyser, PerkinElmer, Boston, MA, USA). Soil available phosphorus was measured by the Olsen method. Soil total phosphorus and plant phosphorus were measured by molybdenum antimony blue colourimetry. Soil hydrolysable nitrogen was measured by alkaline hydrolysis diffusion. Based on the DBH and height of every individual plant, we estimated the biomass of the 54 investigated species of woody plants by the growth equation (Table S1). If some species had no growth equation, we chose the growth equation of a similar species instead^48^. For less frequently occurring species, we chose the growth equation proposed by Ali *et al*.^49^ to estimate the biomass following the suggestion of Lambert *et al*.^50^.

Prior to calculating the metrics of multifunctionality, we first analysed the correlations between the eight functional variables to determine whether they were strongly correlated with one another (Table S2). Among the 28 pairs of relationships, 12 pairs showed significant relationships. However, one exception was noted in that the relationship between soil total carbon and plant nitrogen was slightly negative (*r* = −0.282, *p* < 0.05). Therefore, this finding suggested the lack of no strong trade-offs among the different functions^51^.

### Assessing EMF

We used two complementary approaches to evaluate the role of SR and FRic in driving EMF. The first was the averaging approach^9^. To obtain an averaged multifunctionality index for each site, we first calculated the standardized *Z* scores for the eight variables. The *Z* scores were then averaged to obtain a multifunctionality index^9^.

We next used the multiple threshold approach^10^. Here, we tallied the number of measured functions that simultaneously exceeded multiple critical thresholds, and the threshold was defined as a given percentage of the highest performance for each function. To eliminate potential outliers, the highest performing was defined as an average of the top five values for each function^52^. For our analysis, we selected threshold values ranging from 1 to 99%.

For these two approaches, the averaging approach has been widely used in analyses of multifunctionality^12,28,53^, and the index has good statistical properties. Specifically, it allows us to directly estimate the ability of a community to sustain multiple functions simultaneously^9,21^. It is more suitable for linear model analysis^10^. To date, in the reported approaches, the multiple threshold approach is the most comprehensive method to evaluate BEMF relationships^10,25^. This approach can provide more powerful information and flexibility than other approaches^10^.

### Statistical analyses

We first evaluated the relationships between biodiversity (i.e., SR and FRic) and individual ecosystem functions using Pearson correlation and OLS regression analysis. The relationships among SR, FRic and EMF were evaluated using simple OLS regressions. We then used the multiple threshold method to evaluate the effect of SR and FRic on EMF. *T*_min_ is the minimum threshold where SR or FRic begins to have an effect. *T*_max_ is the threshold beyond which the slope first declined and was not significantly different from zero. *T*_mde_ is the threshold where SR or FRic had its strongest effect. *R*_mde_ is the realized maximum effect of SR and FRic, which is the strength of the linear relationships of SR or FRic-multifunctionality where SR or FRic has the strongest effects. *P*_mde_ is the percentage of the maximum possible SR or FRic effect^10^. According to these parameters, the effect of SR on EMF could be compared with the effect of FRic on EMF^38^. We conducted a multivariate linear regression model to evaluate the comprehensive effects of biotic factors (FRic, SR) and abiotic factors (Soil pH, MAP and MAT) on EMF in the *Pinus yunnanensis* natural secondary forest. To select the best model, we employed different alternative models, such as abiotic factors entered first or species richness entered first into the multivariate linear regression model (Table S3). Then, we used random forest analysis to tease apart the relative importance of these variables on ecosystem multifunctionality. Random forest analysis was used widely because it could determine the effect of each predictor variable individually and could also interact with other predictors in a multivariable manner^54^. Lastly, we used “*impact*” software^55^ to calculate standardized effect size (SES) of functional diversity obtained using null models that control for SR effect and eight individual EFs. SES value = (values_observed_ − mean(values_null_))/sd (values_null_). If the SES value was positive, it would indicate divergence from random. In contrast, if the SES value was negative, it would indicate a random clustering pattern.

All statistical analyses were conducted using R version 3.3.2^56^ (R development Core Team, 2016). FRic was calculated in the ‘FD’ package^57^, and the multiple threshold analysis was performed in the ‘multifunc’ package^10^. The standardized regression coefficients were calculated by package ‘QuantPsyc’ in R. The partial regression coefficients were obtained by Partial Least Squares Regression.

## Supplementary information

Supplementary Information for “Functional diversity drives ecosystem multifunctionality in a Pinus yunnanensis natural secondary forest”
