# Supplementary material for: Functional diversity drives ecosystem multifunctionality in a Pinus yunnanensis natural secondary forest
Source: Sci Rep. 2019 May 6;9:6979. doi: 10.1038/s41598-019-43475-1 (PMC6502788; doi:10.1038/s41598-019-43475-1)
Supplement: Supplementary file 1 — Supplementary Information for “Functional diversity drives ecosystem multifunctionality in a Pinus yunnanensis natural secondary forest” [file 41598_2019_43475_MOESM1_ESM.docx]

Supplementary Information for “Functional diversity drives ecosystem multifunctionality in a *Pinus yunnanensis* natural secondary forest”

Xiaobo Huang^1,2^, Jianrong Su^1,2*^, Shuaifeng Li^1,2^, Wande Liu^1,2^, Xuedong Lang^1,2^

1. Research Institute of Resources Insects, Chinese Academy of Forestry, Kunming 650224, China.

2. Pu’er Forest Ecosystem Research Station, National Forestry and Grassland Administration, Kunming 650224, China.

**Table 1. Equations used in woody plant biomass in *Pinus yunnanensis* natural secondary forest.**

| Woody Plant Biomass Equations | Suitable species |
| --- | --- |
| *W*=534.79(*D^2^H*)^0.9891^+0.001*D*^3.2818^+0.0028*D*^2.4641^+77.449(*D^2^H*)^0.7966 1^ | *Pinus yunnanensis* |
| *W*=0.0245(*D^2^H*)^1.0118 2^ | *Schima superba* |
| *W*=0.027388(*D^2^H*)^0.898869^+0.012101(*D^2^H*)^0.854295^+0.014972(*D^2^H*)^0.875639^+0.010593(*D^2^H*)^0.813953 3^ | *Alnus nepalensis* |
| *W*=0.069(*D^2^H*)^0.888 4^ | *Quercus variabilis* |
| *W*=0.13463*D*^1.97626^*H*^0.48706 5^ | *Quercus aliena*, *Quercus semicarpifolia* |
| *W*=0.0402*D*^2.3108 6^ | *Rhododendron moulmainense*, *Rhododendron decorum*, *Rhododendron delavayi*, *Lyonia ovalifolia*, *Rhododendron spinuliferum*, *Lyonia doyonensis*, *Vaccinium duclouxii*, *Craibiodendron stellatum* |
| Ln(*W*)=-3.67+3.07×Ln(*D*) ^7^ | *Cyclobalanopsis delavayi*, *Cyclobalanopsis kerrii*, *Cyclobalanopsis glaucoides* |
| Ln(*W*)=-3.59+2.47×Ln(*D*) ^7^ | *Eurya groffii* |
| Ln(*W*)=-4.77-0.384×Ln(*D*)+3.72×Ln(*H*) ^7^ | *Symplocos chinensis* |
| *W*=0.04965*D*^2.06846^*H*^0.50563 8^ | *Keteleeria evelyniana* |
| Ln(*W*)=-3.23+2.17×Ln(*D*) ^7^ | *Glochidion lanceolarium*, *Anneslea fragrans*, *Dodonaea viscosa*, *Pyrus pashia*, *Myrica nana*, *Ternstroemia gymnanthera*, *Ficus semicordata*, *Coriaria nepalensis*, *Glochidion eriocarpum*, *Rapanea neriifolia*, *Dichotomanthus tristaniaecarpa*, *Photinia glomerata*, *Osyris wightiana*, *Albizia kalkora*, *Wendlandia uvariifolia*, *Elaeagnus conferta*, *Phyllanthus emblica*, *Ulmus tonkinensis*, *Engelhardtia spicata* |

**References**

1. Sun B. G. *et al*. Biomass Distribution Pattern of Different Parts of *Pinus yunnanensis* with Different Diameter Class. *Forest Research* **25**, 71-76 (2012).
2. Cheng Y., Hong W., Wu C. Z. & Qi X. H. Distribution Characters of *Schima superba* Aboveground Biomass and Its Productivity. *Chin J Appl Environ Biol* **15**, 318-322 (2009).
3. Li G. X., Meng G. T., Fang X. J., Lang N. J., Yuan C. M. & Wen S. L. Characteristics of *Alnus cremastogyne* plantation community and its biomass in central Yunnan Plateau. *Journal of Zhejiang Forestry College* **23**, 362-366 (2006).
4. Zhang Y. A study on the aboveground biomass models of *Quercus variabilis* Blume in Beijing. *Hebei Journal of Forestry and Orchard Research* **26**, 6-8 (2011).
5. Gan S. S., He P. & Xiao Q. H. Establishment of Single-tree Above-ground and Below-ground Biomass Models for Quercus and Birch in the Southwestern China. *Central South Forest Inventory and Planning* **33**, 1-4 (2014).
6. Zhang Q., Li J. X., Xu W. T., Xiong G. M. & Xie Z. Q. Estimation of biomass allocation and carbon density of *Rhododendron simsii* shrubland in the subtropical mountainous areas of China. *Chinese Journal of Plant Ecology* **41**, 43-52 (2017).
7. Ali A *et al*. Allometric biomass equations for shrub and small tree species in subtropical China. *Silva Fennica* **49**, 1-10 (2015).
8. Zeng W. S. Generalized Tree Biomass Equations of Chinese Fir in China. *Central South Forest Inventory and Planning* **32**, 4-11 (2013).

**Table 2.Results of Pearson correlation coefficients between the different variables related to ecosystem functions used to calculate the multifunctionality index.**

|  | PN | PP | SHN | SAP | STN | STP | SOC |
| --- | --- | --- | --- | --- | --- | --- | --- |
| PP | 0.134 |  |  |  |  |  |  |
| SHN | ﹣0.096 | 0.187 |  |  |  |  |  |
| SAP | ﹣0.159 | 0.179 | 0.920** |  |  |  |  |
| STN | ﹣0.216 | 0.557** | 0.258 | 0.424** |  |  |  |
| STP | 0.145 | 0.496** | 0.530** | 0.517** | 0.571** |  |  |
| STC | ﹣0.282* | ﹣0.076 | 0.669** | 0.829** | 0.414** | 0.209 |  |
| WPB | 0.097 | ﹣0.119 | 0.164 | 0.202 | ﹣0.042 | ﹣0.121 | 0.266** |

Significance levels are as follows: **p* ＜ 0.05 and ***p* ＜ 0.01.

**Table 3. The results of multivariate liner regression based on different order of terms entering into the model.**

**No.1**

| Rank | Sum Sq | Mean Sq | F value | Pr (>F) |
| --- | --- | --- | --- | --- |
| SR | 1.9321 | 1.93209 | 6.6762 | 0.012620* |
| FRic | 2.5839 | 2.58390 | 8.9285 | 0.004278** |
| Soil pH | 0.0108 | 0.01077 | 0.0372 | 0.847754 |
| MAP | 0.0652 | 0.06525 | 0.2255 | 0.636896 |
| MAT | 0.00000 | 0.00000 | 0.0000 | 0.997005 |
| ***R^2^=0.2338, Adjusted R^2^=0.1601*** | | | | |

**No.2**

| Rank | Sum Sq | Mean Sq | F value | Pr (>F) |
| --- | --- | --- | --- | --- |
| FRic | 4.0702 | 4.0702 | 14.0645 | 0.0004454 |
| SR | 0.4458 | 0.4458 | 1.5403 | 0.2201480 |
| Soil pH | 0.0108 | 0.0108 | 0.0372 | 0.8477544 |
| MAP | 0.0652 | 0.0652 | 0.2255 | 0.6368962 |
| MAT | 0.0000 | 0.0000 | 0.0000 | 0.9970051 |
| ***R^2^=0.2338, Adjusted R^2^=0.1601*** | | | | |

**No.3**

| Rank | Sum Sq | Mean Sq | F value | Pr (>F) |
| --- | --- | --- | --- | --- |
| SR | 1.9321 | 1.93209 | 6.0707 | 0.01702* |
| Soil pH | 0.1763 | 0.17634 | 0.5541 | 0.45995 |
| MAP | 0.6476 | 0.64762 | 2.0348 | 0.15960 |
| MAT | 0.0165 | 0.01654 | 0.0520 | 0.82056 |
| ***R^2^=0.1412, Adjusted R^2^=0.07635*** | | | | |

**No.4**

| Rank | Sum Sq | Mean Sq | F value | Pr (>F) |
| --- | --- | --- | --- | --- |
| FRic | 4.0702 | 4.0702 | 13.9995 | 0.0004509*** |
| Soil pH | 0.0410 | 0.0410 | 0.1412 | 0.7086327 |
| MAP | 0.1079 | 0.1079 | 0.3712 | 0.5449480 |
| MAT | 0.0122 | 0.0122 | 0.0420 | 0.8384859 |
| ***R^2^=0.2154, Adjusted R^2^=0.1562*** | | | | |

**No.5**

| Rank | Sum Sq | Mean Sq | F value | Pr (>F) |
| --- | --- | --- | --- | --- |
| Soil pH | 0.2284 | 0.22839 | 0.7892 | 0.37844 |
| MAP | 1.5887 | 1.58865 | 5.4895 | 0.02299* |
| MAT | 0.0034 | 0.00342 | 0.0118 | 0.91379 |
| SR | 0.9521 | 0.95213 | 3.2900 | 0.07547• |
| FRic | 1.8194 | 1.81942 | 6.2869 | 0.01533* |
| ***R^2^=0.2338, Adjusted R^2^=0.1601*** | | | | |

**No.6**

| Rank | Sum Sq | Mean Sq | F value | Pr (>F) |
| --- | --- | --- | --- | --- |
| Soil pH | 0.2284 | 0.22839 | 0.7892 | 0.378439 |
| MAP | 1.5887 | 1.58865 | 5.4895 | 0.022992* |
| MAT | 0.0034 | 0.00342 | 0.0118 | 0.913793 |
| FRic | 2.4109 | 2.41094 | 8.3309 | 0.005664** |
| SR | 0.3606 | 0.36061 | 1.2461 | 0.269436 |
| ***R^2^=0.2338, Adjusted R^2^=0.1601*** | | | | |

Significance levels are as follows: **P* ＜ 0.05 and ***P* ＜ 0.01.
